# Monitoring Patients With Implantable Cardioverter Defibrillators Using Mobile Phone Electrocardiogram: Case Study

**DOI:** 10.2196/cardio.8710

**Published:** 2018-02-21

**Authors:** Caley Kropp, Jordan Ellis, Rajasekhar Nekkanti, Samuel Sears

**Affiliations:** ^1^ Department of Psychology East Carolina University Greenville, NC United States; ^2^ Department of Cardiovascular Sciences East Carolina University Greenville, NC United States

**Keywords:** atrial fibrillation, ICD, ECG, mobile phone monitoring, mobile health, electrophysiology

## Abstract

**Background:**

Preventable poor health outcomes associated with atrial fibrillation continue to make early detection a priority. A one-lead mobile electrocardiogram (mECG) device given to patients with an implantable cardioverter defibrillator (ICD) allowed users to receive real-time ECG readings in 30 seconds.

**Objective:**

Three cases were selected from an institutional review board-approved clinical trial aimed at assessing mECG device usage and satisfaction, patient engagement, quality of life (QoL), and cardiac anxiety. These three specific cases were selected to examine a variety of possible patient presentations and user experiences.

**Methods:**

Three ICD patients with mobile phones who were being seen in an adult device clinic were asked to participate. The participants chosen represented individuals with varying degrees of reported education and patient engagement. Participants were instructed to use the mECG device at least once per day for 30 days. Positive ECGs for atrial fibrillation were evaluated in clinic. At follow-up, information was collected regarding their frequency of use of the mECG device and three psychological outcomes in the domains of patient engagement, QoL, and cardiac anxiety.

**Results:**

Each patient used the technology approximately daily or every other day as prescribed. At the 30-day follow-up, usage reports indicated an average of 32 readings per month per participant. At 90-day follow-up, usage reports indicated an average of 34 readings per month per participant. Two of the three participants self-reported a significant improvement in their physical QoL from baseline to completion, while simultaneously self-reporting a significant decrease in their mental QoL. All three participants reported high levels of device acceptance and technology satisfaction.

**Conclusions:**

This case study demonstrates that ICD patients with varying degrees of education and patient engagement were relatively active in their use of mECGs. All three participants using the mECG technology reported high technology satisfaction and device acceptance. High sensitivity, specificity, and accuracy of mECG technology may allow routine atrial fibrillation screening at lower costs, in addition to improving patient outcomes.

## Introduction

### Background

Implantable cardioverter defibrillators (ICDs) have demonstrated a mortality advantage in randomized clinical trials compared with usual care and antiarrhythmic drug treatments in at-risk patients [1]. The full suite of diagnostic capabilities of ICDs, such as detection of atrial arrhythmias, provide additional value to health care professionals. However, patients with ICDs have not had equal access to this information. Recently, multiple consumer products have been approved for cardiac monitoring, including mobile phone-based systems that provide physician-interpreted electrocardiograms (ECGs) on demand. The overall impact and value of engaging patients in the use of these services continues to emerge, but the utility of potentially detecting the initiation of atrial fibrillation could be significant.

Atrial fibrillation is the most common cardiac arrhythmia worldwide and continues to be a major burden to public health [2]. It affects up to 6 million American adults, which is expected to double over the next 25 years [2]. Furthermore, atrial fibrillation is associated with a five-times increased risk of stroke [2] and a three-times increased risk of heart failure [3]. Approximately one of three patients with atrial fibrillation have “silent” or underdetected symptoms, highlighting the potential importance of intermittent ECG data acquisition [4]. Many of the associated poor outcomes of atrial fibrillation are thought to be highly preventable, making early identification of atrial fibrillation a priority issue.

### Patient Engagement and Self-Management

Patient engagement refers to the attitudes and behaviors of patients, and the ways in which they interact with their own health care management plans [5,6]. Research has shown that patients who are more engaged in managing their health care needs may yield more positive clinical outcomes than their less-engaged peers [5,7]. Positive patient engagement helps promote good health behaviors and can increase overall life satisfaction [5,8]. Due to advances in technology, patients are now increasing their patient engagement and self-management through the use of health-related mobile phone-based apps.

### KardiaMobile by AliveCor

For ICD patients, using a KardiaMobile by AliveCor, Inc [9] device with the associated Kardia app has been one way that patients are able to increase their patient engagement. The KardiaMobile mobile ECG (mECG) device is one of many recently developed, noninvasive diagnostic tools. Although using these devices does not replace the need for regularly scheduled 12-lead ECG readings, more frequent screening allows patients to play a more substantial role in their health care. The KardiaMobile mECG device is half the size of a credit card and can securely attach to the back of a mobile phone or tablet. This one-lead device is cleared by the US Food and Drug Administration and can accurately detect atrial fibrillation in 30 seconds [4]. The instantaneous analysis of the user’s ECG reports three different outcomes: “normal,” “possible atrial fibrillation,” and “unclassified.” The development of mobile-supported health apps may allow patients and their health care providers to use medical information to improve patient satisfaction and health security, reduce costs, and improve health outcomes. However, no research to date has investigated usage and satisfaction with the KardiaMobile device, or the psychosocial correlates. The purpose of this case study is to examine the utility and impact of mobile phone-based ECG readings in three ICD patients who are enrolled in a clinical study.

## Methods

Patients with ICDs were approached for study participation at their regularly occurring device-check appointments. Three unique cases were selected from an institutional review board-approved clinical trial aimed at assessing KardiaMobile device usage and satisfaction, patient engagement, QoL, and cardiac anxiety. The three cases chosen were selected to examine a variety of possible patient presentations and user experiences. Participants were not compensated for their participation, but they were allowed to keep their KardiaMobile devices free of charge at the conclusion of the study period.

The first participant was selected because they reported a high percentage of atrial fibrillation readings in comparison to other patients. The second participant was selected to include a participant who reported above average QoL scores and high overall KardiaMobile device usage. The third participant was included because they reported low baseline QoL in both the physical and mental health domains.

Patients were administered the Cardiac Anxiety Questionnaire (CAQ), which is an 18-item self-report measure designed to assess cardiac anxiety [10]. Higher mean scores indicate greater cardiac anxiety symptoms. Participants also completed the Short Form Health Survey version 2 (SF-12v2), a 12-item questionnaire used to measure functional health and well-being from a patient perspective [11]. These measures were administered at baseline and at 30- and 90-day follow-ups. The SF-12v2 provides two summary scores of QoL: a mental health subscale and a physical health subscale (higher scores indicate greater QoL). KardiaMobile usage reports were also collected at the 30- and 90-day follow-up research appointments. Patients were asked at the 30-day follow-up if they would like to continue using the KardiaMobile device. Additionally, patients self-reported on an item which stated, “I am satisfied with my use of the KardiaMobile device” and responded on a 5-point Likert scale from “strongly disagree” to “strongly agree,” at each follow-up administration.

## Results

### Participant 1

**Figure 1:**
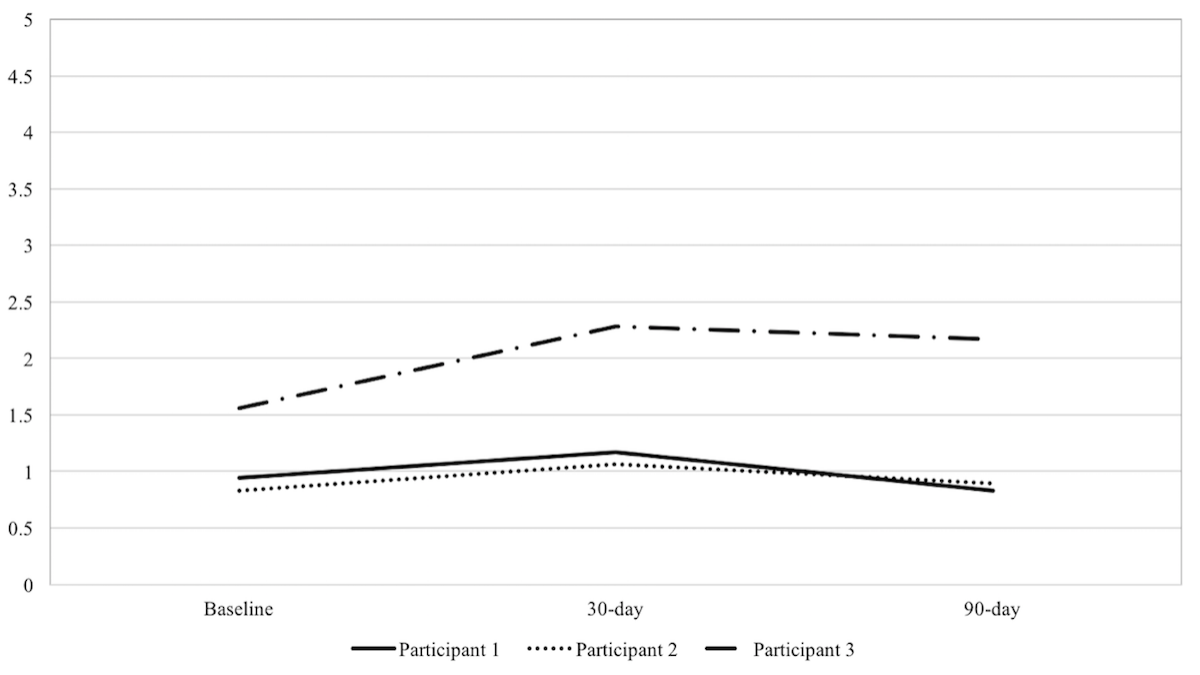
Cardiac Anxiety Questionnaire scores at baseline and at 30- and 90-day follow-ups (N=3).

The first participant was a married, white woman (age approximately 60 years), who held a graduate degree and reported an annual income of US $50,000 to US $74,000. Her cardiac medical history included diagnoses of atrial fibrillation, congestive heart failure, ventricular tachycardia, and nonischemic cardiomyopathy. Her KardiaMobile usage reports indicated that at 30-day follow-up she had used her device 34 times (0%, 0/34 normal readings; 59%, 20/34 atrial fibrillation readings; 41%, 14/34 unclassified readings). She agreed to continue using the device for an additional 60-day period, and at the 90-day follow-up she had used the device a total of 109 times (3.7%, 4/109 normal readings; 77.1%, 84/109 atrial fibrillation readings; 19.3%, 21/109 unclassified readings). As shown in Table 1, participant 1 reported very strong agreement to being satisfied with use of the device at both 30-day and 90-day follow-ups. Participant 1 reported average mental and physical well-being across all time points. She also reported below average cardiac anxiety across all time points (see Figure 1).

### Participant 2

The second participant was a married, white male (age approximately 70 years) who had completed some college and reported an annual income of US $30,000 to US $39,999. His medical history included atrial fibrillation, coronary artery disease, hypertension, sustained ventricular tachycardia, and Twiddler’s syndrome. KardiaMobile usage reports indicated that at 30-day follow-up he had used his device 37 times (89%, 33/37 normal readings; 5%, 2/37 atrial fibrillaton readings; 5%, 2/37 unclassified readings). He agreed to continue using the device for an additional 60-day period, and at the 90-day follow-up he had used the device a total of 139 times (88.5%, 123/139 normal readings; 9.4%, 13/139 atrial fibrillation readings; 2.2%, 3/139 unclassified readings). Participant 2 agreed very strongly to being satisfied with use of the device at both 30-day and 90-day follow-ups. Participant 2 reported high mental well-being and low physical well-being at baseline (see Table 1). His reported physical QoL increased significantly from baseline to 30-day follow-up. His mental well-being dropped slightly over time; however, his score continued to suggest good mental QoL. His score on the CAQ indicated below average cardiac anxiety, and his score remained stable across time points (see Figure 1).

**Table 1:** Outcomes following KardiaMobile usage in three patients with implantable cardioverter defibrillators.

Outcomes		Participant 1	Participant 2	Participant 3
**Usage (30-day), n**				
	Total	34	37	25
	Normal	0	33	23
	Atrial fibrillation	20	2	0
	Unclassified	14	2	2
**Usage (90-day), n**				
	Total	109	139	61
	Normal	4	123	56
	Atrial fibrillation	84	13	2
	Unclassified	21	3	3
**CAQ** ^a^ **, score**				
	Baseline	0.94	0.83	1.56
	30-day	1.17	1.06	2.28
	90-day	0.83	0.89	2.17
**SF-12v2 Physical** ^b^ **, score**				
	Baseline	57	22	28
	30-day	55	44^c^	31
	90-day	54	35^c^	34^c^
**SF-12v2 Mental** ^b^ **, score**				
	Baseline	56	72	24
	30-day	58	65^c^	23
	90-day	58	67	18^c^

^a^CAQ: Cardiac Anxiety Questionnaire. The development paper of the CAQ reported a mean total score of 1.67 (SD 0.81) in a sample of 42 cardiac patients [10].

^b^SF-12v2: 12-Item Short Form Survey version 2. Mean scores on both SF-12 subscales were 50 (SD 10).

^c^Indicates a significant change (5 points) on an SF-12v2 subscale.

### Participant 3

The third participant was a married, African-American male (age approximately 60 years), who had completed a high school degree and reported an annual income of US $30,000 to US $39,999. His cardiac medical history included myocardial infarction, peripheral artery disease, ischemic cardiomyopathy, and hypertension. KardiaMobile usage reports indicated that at 30-day follow-up he had used his device 25 times (92%, 23/25 normal readings; 0%, 0/25 atrial fibrillation readings; 8%, 2/25 unclassified readings). He agreed to continue using the device for an additional 60-day period, and at the 90-day follow-up he had used the device a total of 61 times (92%, 56/61 normal readings; 3%, 2/61 atrial fibrillation readings; 5%, 3/61 unclassified). Participant 3 reported very strong agreement to being satisfied with use of the device at both 30-day and 90-day follow-ups. Participant 3 reported low QoL in both the physical health and mental health domains, in comparison to SF-12v2 norms for US adults, across all time points (see Table 1). Participant 3’s physical QoL slightly improved from baseline to 90-day follow-up; however, he also reported a slight decline in mental well-being during this time period. He reported high cardiac anxiety, which increased from baseline to 30-day follow-up. In comparison to the CAQ development norms [12], his score at 30-day follow-up fell to the 80th percentile (see Figure 1).

## Discussion

The current case series demonstrated that ICD patients with varying degrees of education and patient engagement were relatively active in their use of mobile phone-based ECGs. At baseline, patients were asked to use the KardiaMobile device at least once per day. Across patients presented, each patient used the technology approximately daily or every other day. At the 30-day follow-up, usage reports indicated an average of 32 readings per month per participant. At 90-day follow-up, usage reports indicated an average of 34 readings per month per participant. All three participants exhibited high levels of device acceptance, but the appraisals associated with usage is likely to be different for each individual user. Patients with ICDs may benefit from increased access to cardiac technical information because it is available to them, or patients may feel overburdened. For instance, cardiac anxiety, which was elevated in participant 3, is associated with excessive symptom monitoring, and increased access through mobile monitoring could maintain problematic checking behaviors and other symptoms of anxiety in some patients [13]. However, patients who experience cardiac anxiety maintained by the avoidance of heart-related stimuli could benefit from exposure related to mobile monitoring. Nonetheless, patient engagement in this new technology appears to be high and associated with limited negative effects.

Other psychological factors associated with KardiaMobile usage should be explored further. High usage may be be driven by the novelty of the product and positive feelings associated with the ability to self-monitor (ie, posivite reinforcement). However, high usage in some individuals could be driven by negative reinforcement (ie, when a behavior is strengthened because it provides escape from aversive stimuli) through excessive reassurance-seeking behaviors. Excessive reassurance seeking (eg, excessively seeking attention of family members to physiological symptoms due to fear of dysfunction) provides feelings of relief in the short term, but may sustain fears and anxiety about health in the long term [10]. KardiaMobile use and other patient-centered technologies have the potential to serve as a real-time reassurance mechanism, but providers should pay attention to problematic excessive use patterns that could be maintaining health anxiety.

There were several limitations associated with the current study. Patients with ICDs are inherently reliant on their implantable devices due to the nature of their disease state, which may have increased mECG device acceptance. Additionally, the opt-in nature of the research may have led to an overestimation of technology satisfaction. Although KardiaMobile has been clinically validated for general population use, the overall utility of mECG devices is largely unknown in patients with diagnosed conditions. It is likely that patients with known symptoms would have increased motivation for symptom monitoring. However, the mECG device used in this study has been validated as a diagnostic tool, but its clinical utility for continued management and monitoring of treatment effect has not been investigated. Mobile ECG devices have been marketed toward users with previously undetected symptoms, and “silent” sypmtoms of atrial fibrillation occur in approximately one in three patients with atrial fibrillation [4].

Patients are increasingly being asked to be key shareholders of their health care teams, and condition-specific medical technology is now allowing patients to monitor signs and symptoms from the comfort of their own homes. KardiaMobile is one example of how the patient engagement movement is allowing users to become more involved by putting ECG technology in the hands of the person most affected. However, hypervigilance and checking behaviors have been shown to be associated with an inflated sense of responsibility, which is a potential risk for mECG users [14]. Although it is still unclear whether or not this novel technology has positive or negative effects on cardiac-related anxiety or physical/mental health components, the most significant findings of this report show that despite a number of differences in background and presentation of illness, all three users reported high levels of technology satisfaction using the KardiaMobile device. Ongoing registry research will provide additional information.

Finally, the effect of increased patient engagement with specific medical technology on physician well-being should be considered because these devices could be perceived as an additional burden. Driven by ever-increasing expectations and responsibilities, occupational burnout rates are high among cardiologists, and successful mitigation of burnout will require adaptations by providers, patients, and health care systems [15]. Critical evaluation of this technology by patients and providers is needed, and the consequences related to tasking patients with data acquisition and symptom interpretation requires thoughtful consideration before clinical implementation. Employment of new technology will be most successful when providers are able to see these adaptations not as additional clinical duties, but as part of their overall mission to provide patient-centered care.

The KardiaMobile device by AliveCor is a novel way for cardiac patients to monitor and track their own ECG recordings and share them with their medical providers. This technology may not be indicated for all patients, especially for users with preexisting cardiac-related anxiety. However, there is preliminary data to suggest that many users would have high technology satisfaction using the device. As advances in mobile technology continue to evolve the landscape of health care, ICD patients are encouraged to work collaboratively with their providers to answer the question, “Is smartphone ECG technology the ‘smart’ option for me?”

## Abbreviations

CAQ: Cardiac Anxiety Questionnaire
ECG: electrocardiogram
ICD: implantable cardioverter defibrillator
mECG: mobile electrocardiogram
QoL: quality of life
SF-12v2: 12-Item Short Form Survey version 2

## References

[1] Bardy G, Lee K, Mark D, Poole JE, Packer DL, Boineau R, Domanski M, Troutman C, Anderson J, Johnson G, McNulty SE, Clapp-Channing N, Davidson-Ray LD, Fraulo ES, Fishbein DP, Luceri RM, Ip JH, Sudden Cardiac Death in Heart Failure Trial (SCD-HeFT) Investigators (2005). Amiodarone or an implantable cardioverter-defibrillator for congestive heart failure. N Engl J Med.

[2] Lowres N, Redfern J, Freedman SB, Orchard J, Bennett AA, Briffa T, Bauman A, Neubeck L (2014). Choice of Health Options In prevention of Cardiovascular Events for people with Atrial Fibrillation (CHOICE-AF): A pilot study. Eur J Cardiovasc Nur.

[3] Stewart S, Hart CL, Hole DJ, McMurray JJ (2002). A population-based study of the long-term risks associated with atrial fibrillation: 20-year follow-up of the Renfrew/Paisley study. Am J Med.

[4] Williams J, Pearce K, Bennet I, Williams J, Manchester M (2015). The effectiveness of a mobile ECG device in identifying AF: sensitivity, specificity and predictive value. Br J Cardiol.

[5] Barello S, Triberti S, Graffigna G, Libreri C, Serino S, Hibbard J, Riva G (2015). eHealth for patient engagement: a systematic review. Front Psychol.

[6] Menichetti J, Libreri C, Lozza E, Graffigna G (2016). Giving patients a starring role in their own care: a bibliometric analysis of the on-going literature debate. Health Expect.

[7] Greene J, Hibbard JH (2012). Why does patient activation matter? An examination of the relationships between patient activation and health-related outcomes. J Gen Intern Med.

[8] Barello S, Graffigna G (2015). Patient engagement in healthcare: pathways for effective medical decision making. Neuropsychol Trends.

[9] AliveCor.

[10] Eifert GH, Thompson RN, Zvolensky MJ, Edwards K, Frazer NL, Haddad JW, Davig J (2000). The cardiac anxiety questionnaire: development and preliminary validity. Behav Res Ther.

[11] Ware J, Kosinski M, Keller SD (1996). A 12-Item Short-Form Health Survey: construction of scales and preliminary tests of reliability and validity. Med Care.

[12] Halldorsson B, Salkovskis P (2017). Why do people with OCD and health anxiety seek reassurance excessively? An investigation of differences and similarities in function. Cognit Ther Res.

[13] Aikens JE, Zvolensky MJ, Eifert GH (2001). Differential fear of cardiopulmonary sensations in emergency room noncardiac chest pain patients. J Behav Med.

[14] Belayachi S, Van der Linden M (2017). The cognitive heterogeneity of obsessive-compulsive checking. J Cognit Educat Psychol.

[15] Michel J, Sangha D, Erwin J (2017). Burnout among cardiologists. Am J Cardiol.

